# The Impact of Maternal Obesity on Human Milk Macronutrient Composition: A Systematic Review and Meta-Analysis

**DOI:** 10.3390/nu12040934

**Published:** 2020-03-27

**Authors:** Gabriela E. Leghi, Merryn J. Netting, Philippa F. Middleton, Mary E. Wlodek, Donna T. Geddes, Beverly S. Muhlhausler

**Affiliations:** 1 School of Agriculture, Food and Wine, The University of Adelaide, Adelaide, SA 5064, Australia; gabriela.estevesleghi@adelaide.edu.au; 2 Women and Kids Theme, South Australian Health and Medical Research Institute (SAHMRI), Adelaide, SA 5000, Australia; merryn.netting@sahmri.com (M.J.N.); philippa.middleton@sahmri.com (P.F.M.); 3 Discipline of Paediatrics, The University of Adelaide, Adelaide, SA 5000, Australia; 4 Department of Physiology, The University of Melbourne, Melbourne, VIC 3010, Australia; m.wlodek@unimelb.edu.au; 5 School of Molecular Sciences, The University of Western Australia, Perth, WA 6009, Australia; donna.geddes@uwa.edu.au; 6 Commonwealth Scientific and Industrial Research Organisation (CSIRO), Adelaide, SA 5000, Australia

**Keywords:** Systematic review, maternal obesity, body mass index (BMI), adiposity, human milk composition, macronutrient, infant health

## Abstract

Maternal obesity has been associated with changes in the macronutrient concentration of human milk (HM), which have the potential to promote weight gain and increase the long-term risk of obesity in the infant. This article aimed to provide a synthesis of studies evaluating the effects of maternal overweight and obesity on the concentrations of macronutrients in HM. EMBASE, MEDLINE/PubMed, Cochrane Library, Scopus, Web of Science, and ProQuest databases were searched for relevant articles. Two authors conducted screening, data extraction, and quality assessment independently. A total of 31 studies (5078 lactating women) were included in the qualitative synthesis and nine studies (872 lactating women) in the quantitative synthesis. Overall, maternal body mass index (BMI) and adiposity measurements were associated with higher HM fat and lactose concentrations at different stages of lactation, whereas protein concentration in HM did not appear to differ between overweight and/or obese and normal weight women. However, given the considerable variability in the results between studies and low quality of many of the included studies, further research is needed to establish the impact of maternal overweight and obesity on HM composition. This is particularly relevant considering potential implications of higher HM fat concentration on both growth and fat deposition during the first few months of infancy and long-term risk of obesity.

## 1. Introduction

The World Health Organization (WHO) defines overweight as a body mass index (BMI) greater than or equal to 25 kg/m^2^ and obesity as a BMI equal or greater than 30 kg/m^2^ [1]. The number of women who are overweight or obese while pregnant and breastfeeding has increased markedly in the past few decades, in line with the global increase in obesity rates [2]. In Australia, around 50% of pregnant women are overweight or obese when attending their first antenatal appointment [3,4], and similar statistics have been reported in the USA and UK [5]. The increasing body weights of pregnant and breastfeeding women has led to a growing interest in the consequences of maternal overweight and obesity for both pregnancy outcomes and infant and child health [6,7].

There are increasing suggestions that, in addition to increasing the risk of pregnancy and neonatal complications, maternal overweight and obesity may be associated with changes in the macronutrient composition of human milk (HM) [8,9,10]. This is of clinical relevance, given the increasing number of studies that have reported associations between the levels of specific HM components, including fat, protein, and lactose in HM, as well as growth and fat deposition in the infant in the first 12 months of age [11,12]. Thus, changes in HM composition have the potential to promote weight gain and fat deposition in the infant [11], and thereby increase their risk of obesity and metabolic disease later in life. The global increase in the incidence of overweight and obesity has raised concerns about the impact this may be having on contemporary HM composition at a population level, and the potential for these compositional changes to be contributing to the current intergenerational cycle of obesity.

While a previous systematic review examined the effect of maternal overweight and obesity on levels of metabolic hormones in HM [13], there have been no studies to date that have systematically synthesized the evidence regarding the effect of maternal overweight and obesity on HM macronutrient composition. Therefore, the purpose of this systematic review and meta-analysis was to undertake a synthesis of studies evaluating the effects of maternal obesity, including BMI and other measures of adiposity, on the concentrations of macronutrients in HM.

## 2. Materials and Methods

### 2.1. Protocol

The present systematic review protocol was developed based on Preferred Reporting Items for Systematic Reviews and Meta-Analysis Protocols (PRISMA-P) guidelines [14] and Cochrane Handbook for Systematic Reviews of Interventions [15].

### 2.2. Eligibility Criteria

To be eligible for inclusion, studies had to include overweight and/or obese women who were either breastfeeding (either exclusively or not) or routinely expressing HM (manually or using a breast pump) and a measure of at least one macronutrient (fat, protein, or lactose) in HM. The studies had to state the time postpartum when HM was collected (to enable stage of lactation to be determined) and to report at least one measure of maternal obesity (either self-reported or obtained by study staff) including body mass index (BMI), skinfold thickness, and bioelectrical impedance analysis (BIA), as well as information regarding when the measurement was performed (e.g., pre-pregnancy, at the time of milk collection etc.). Studies only reported as abstracts or not available in English were excluded. 

### 2.3. Search Strategy

The EMBASE, MEDLINE/PubMed, Cochrane Library, Scopus, Web of Science, ProQuest Dissertations and Thesis Global databases were searched. The search terms, medical subject headings (MeSH) terms and truncation symbol (*) used for MEDLINE/PubMed were (milk, human (MeSH) OR human milk* OR breast milk* OR breastmilk* OR lactation) OR (breast feeding (MeSH) OR breast milk express* OR breastmilk express*) AND (body mass index (MeSH) OR overweight (MeSH) OR obesity (MeSH) OR body composition (MeSH)) AND female (MeSH). The search strategy was adapted to the subject headings and syntax of the other electronic databases. The literature search was limited to studies in humans, but no date range restrictions were applied. Reference lists of included studies were scanned for potentially relevant articles. The last search was conducted in December 2018. 

### 2.4. Selection Process

The search results were initially uploaded into EndNote software [16] and, after removal of duplicates, transferred to the Covidence system [17]. The selection of articles for inclusion in the review was undertaken in two stages. The first stage involved screening the title and abstracts of the search results against the eligibility criteria. In the second stage, the full articles of papers selected in the title/abstract screening stage were screened to confirm that they met the eligibility criteria. At both stages, each article was screened independently by two authors. Disagreement in eligibility status between the first two authors were resolved by a third author or mutual discussion.

### 2.5. Data Extraction

Two authors independently extracted data from each study based on a standardized extraction form adapted from the Cochrane Pregnancy and Childbirth Group [18]. Data extracted included details of study design, participants (sample size, country/region, stage of lactation, whether infants were term or preterm, and whether women were or were not exclusively breastfeeding), details of HM collection methodology (whether pre-feed, post-feed or full expression HM was collected, time of collection, mode of collection, volume, time since last feed/expression, breast used for collection, and whether mother was fasted before collection), measure of maternal obesity (method and time of measurement), and outcomes (HM macronutrient concentration and analytical method). In the case of missing data, reasonable efforts were made to contact the corresponding authors by email. For the purpose of performing meta-analyses, macronutrient concentrations were converted to g/L. Mean and standard deviations for each macronutrient were averaged into single values, respectively, in case of more than one HM value reported for each lactation stage (colostrum, transitional, and mature). Colostrum was defined as milk produced until four days postpartum, transitional milk as milk between four days and two weeks postpartum, and as mature milk thereafter [19].

### 2.6. Quality Assessment of Individual Studies

Quality assessment was based on The Newcastle–Ottawa Scale (NOS), designed for assessing non-randomized studies [20]. Reporting details were based on a pragmatic score reported by Andreas et al. (2014) [13], and conducted independently by two authors. The NOS assessed the representativeness of the cohort (“truly represents” and “somewhat represents” the average lactating women in the community), ascertainment of exposure (whether maternal weight/fat mass was measured by study staff or obtained from medical records) and whether the study controlled for any confounding factors (e.g., maternal and infant age, infant sex). We also included additional categories: sample size (small = studies with < 50 participants, medium = studies with between 50–100 participants, and large = studies with > 100 participants); and whether the study (i) stated gestational age of infants, (ii) stated feeding mode (exclusively or partially breastfeeding), (iii) standardized the time when milk sample/s were collected, and (iv) stated method of HM collection (e.g., full expression or pre- and/or post-feed). One other category, whether study included or controlled for maternal pre-existing chronic conditions, such as gestational diabetes mellitus (GDM) and diabetes, was also included in the quality assessment but these data are presented separately since they were not relevant for all included studies.

### 2.7. Data Synthesis

Findings are presented as structured tables, followed by a description and discussion of the study characteristics that may affect the cumulative evidence. To be eligible for the quantitative synthesis, studies had to report a comparison of HM macronutrient concentrations between normal weight and overweight/obese women following the BMI classification described by the WHO and the standard deviation or other measures of variability for their data. Meta-analyses of eligible studies were conducted using Review Manager software version 5.3. Results are presented by lactation stage (colostrum, transitional, and mature milk) and grouped by overweight and obese women compared to women of normal weight. Data are presented as forest plots, including pooled mean, standard deviation, and 95% confidence intervals. A fixed-effects model was applied, however, if there was a high level of heterogeneity (I^2^ > 50%) between studies a random-effects model was applied. For the purpose of this systematic review, carbohydrate concentration was considered to be equivalent to lactose, and triglyceride levels were considered to be equivalent to fat, since lactose makes up approximately 98% of the total carbohydrates and triglyceride makes up 98% of the total lipids in HM [21,22].

## 3. Results

### 3.1. Summary of Studies

**Figure 1:**
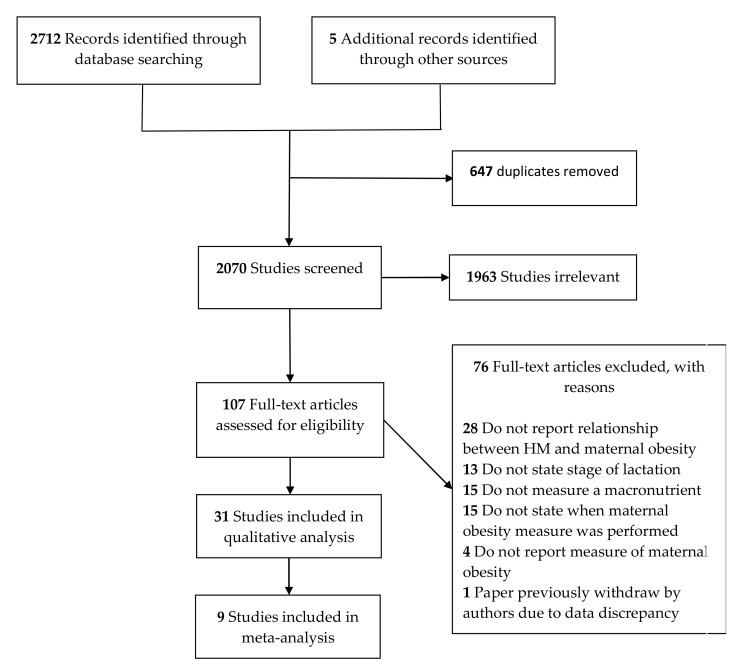
Preferred Reporting Items for Systematic Reviews and Meta-Analysis (PRISMA) flow diagram highlighting the process of article screening and reasons to exclude.

The search strategy identified 2712 articles, with 5 additional publications identified through reference lists, providing a total of 2717 studies. After removal of duplicates and screening of titles/abstracts and full text, 31 studies were included in the qualitative synthesis (5078 lactating women). Of these, nine studies were eligible for inclusion in the meta-analysis (872 lactating women). The remaining 22 studies either did not undertake a comparison between normal weight and overweight and/or obese women (*n* = 15), did not provide measures of variability for their data (*n* = 3), did not report values for transitional and mature milk separately (*n* = 1) or did not report or follow the BMI classification system described by the WHO (*n* = 3). A Preferred Reporting Items for Systematic Reviews and Meta-Analysis (PRISMA) flow diagram is presented in Figure 1.

A summary of included studies is presented in Table 1. The studies included in this review had a broad range of publication dates, between 1986 and 2018, however, most were published after 2000 (*n* = 27). Studies were conducted in 20 different countries/regions with eight studies conducted in the USA. Most studies were cross-sectional (*n* = 16), followed by longitudinal (*n* = 13), and interventional (*n* = 2) in design. Sample sizes ranged from 13 to 2632 women, with the largest studies conducted in Korea and China (*n* = 436 women). Most of the studies collected mature HM samples (*n* = 27), followed by transitional HM (*n* = 8), and colostrum samples (*n* = 7). Besides measures of maternal BMI, eight studies also collected other measures of maternal adiposity, including skinfold and body composition (fat and lean mass). HM fat concentration was reported in 28 of the 31 studies, protein concentrations were reported in 23 studies, while carbohydrate/lactose concentrations were reported in 19 studies.

A summary of all studies reporting comparisons of HM macronutrient concentrations between overweight and/or obese and normal weight women and associations between maternal BMI and/or other measures of obesity and macronutrient concentrations in their milk is presented in Table 2. A summary of results for the qualitative synthesis across all stages of lactation is presented in Table 3. 

### 3.2. Fat Concentration 

**Figure 2:**
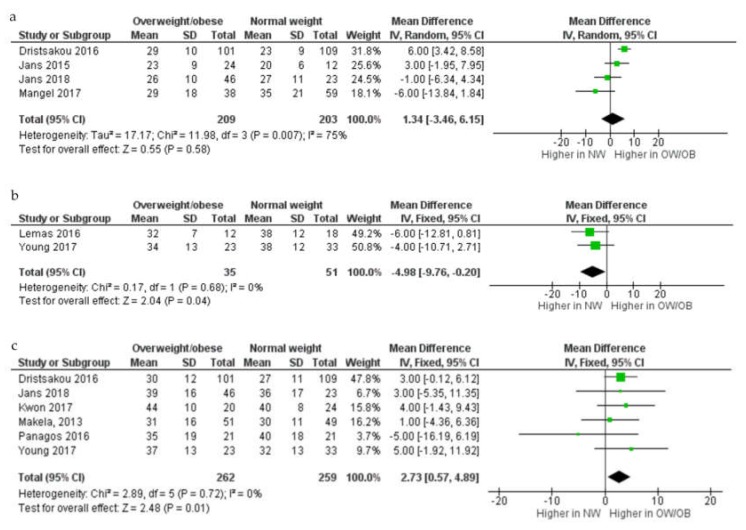
Comparison of fat concentration (g/L) from normal weight and overweight and obese women according to stage of lactation, colostrum (**a**), transitional (**b**) and mature (**c**). CI, confidence interval; Chi^2^ and I^2^, measures of heterogeneity; IV, inverse variance; NW, normal weight women; OB, obese women; OW, overweight women; SD, standard deviation.

In the meta-analysis (*n* = 9; Figure 2), no difference in fat concentration in colostrum between overweight and obese and normal weight women was seen (mean difference (MD) 1.34 g/L, 95% confidence interval (CI) –3.46–6.15, random-effects model, *p* = 0.58; I^2^ = 75%; four studies; 209 overweight and obese women and 203 normal weight women)). In transitional milk, however, fat concentration (g/L) was lower in overweight and obese women compared to those of normal weight (MD –4.98g/L, 95% CI –9.76–0.20, two studies; 35 overweight and obese and 51 normal weight women). In mature milk, fat concentration (g/L) was higher in mature HM from overweight and obese women compared to normal weight women MD 2.73 g/L, 95% CI 0.57–4.89, six studies; 262 overweight and obese women and 259 normal weight women). 

Of the 19 studies that were not eligible for inclusion in the meta-analysis, seven reported a comparison of fat concentrations between normal weight and overweight and/or obese women while 13 assessed associations between HM fat content and one or more measures of maternal obesity (one study reported both comparison and association).

Six of the seven studies that reported comparisons were conducted in mature milk. Of these one found that HM fat concentration was higher in overweight, but not obese, women [45]; one reported higher fat concentrations in women with a BMI ≥23 kg/m^2^ [26]; one found higher fat concentrations in women with higher arm circumference and triceps skinfold thickness measurement [27]; and three found no difference in HM fat concentration between overweight and/or obese and normal women [23,29,48]. Of the two studies conducted in transitional milk, one reported a higher HM fat concentration in overweight women [45] while the other found no difference [23]. The study assessing colostrum samples reported a higher fat concentration in obese women compared to those of normal weight and overweight [32].

Of the 13 studies that assessed associations between HM fat concentration and milk composition, 12 assessed these relationships in mature HM, three in transitional HM, two in colostrum, and two included multiple stages and did not distinguish between them (five studies reported associations for more than one HM type). Eight of the 12 studies in mature milk reported a positive association between women’s BMI and/or percentage body fat and fat levels in their milk [8,26,28,36,43,46,47,49], while four found no association [24,31,37,40]. All three studies conducted in transitional HM reported a positive association between maternal BMI and HM fat concentration [8,36,49]. None of the two studies that assessed levels in colostrum found any association with maternal BMI [8,31]. In agreement, Ley 2012 found no association between maternal BMI and fat concentrations in either colostrum or transitional HM [40]. Quinn 2012, which included samples assessed over multiple stages of lactation, also found no association between maternal BMI or percentage body fat and fat concentrations in HM [44].

### 3.3. Protein Concentration

**Figure 3:**
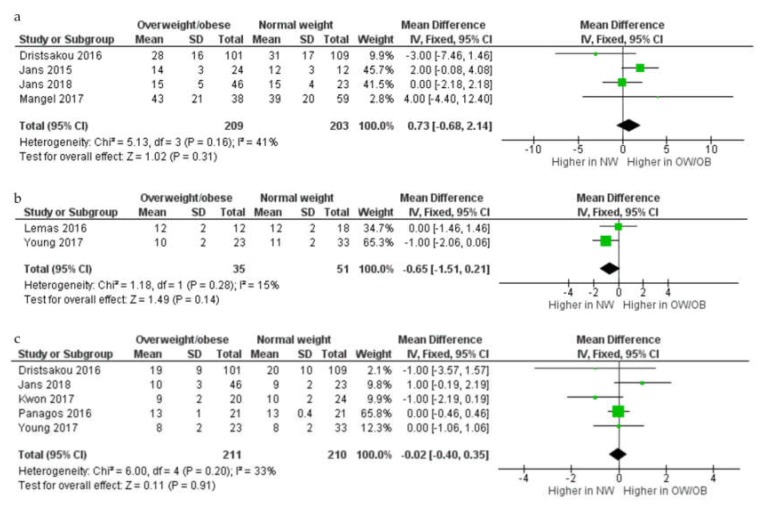
Comparison of protein concentration (g/L) from normal weight and overweight and obese women according to stage of lactation, colostrum (**a**), transitional (**b**), and mature (**c**). CI, confidence interval; Chi^2^ and I^2^, measures of heterogeneity; IV, inverse variance; NW, normal weight women; OB, obese women; OW, overweight women; SD, standard deviation.

In the meta analyses (*n* = 8; Figure 3), there was no difference in protein concentration (g/L) in HM between overweight and obese and normal weight women in either colostrum (MD 0.73 g/L, 95% CI –0.68–2.14,; four studies; 209 overweight and obese women and 203 normal weight women), transitional (MD –0.65 g/L, 95% CI –1.51–0.21, two studies; 35 overweight and obese women and 51 normal weight women), or mature HM (MD –0.02 g/L, 95% CI –0.40–0.35, fixed-effects model, *p* = 0.91; I^2^ = 33%; five studies; 211 overweight and obese women and 210 normal weight women).

All five studies not eligible for inclusion in the meta-analysis that compared HM protein concentration between normal weight and overweight and/or obese women found no differences in HM protein concentration between these groups [23,26,27,29,32], although this included one study that considered >23 kg/m^2^ as overweight and obese [26]. This was independent of the stage of lactation when the samples were collected (*n* = 1 colostrum, *n* = 1 transitional HM, *n* = 4 mature HM; one study reported comparison for more than one HM type).

Of the 11 studies that reported correlations between HM protein concentration and maternal BMI and/or fat mass, 10 examined these associations in mature HM, two in transitional HM, two in colostrum, one in either transitional or colostrum, and one without clear distinction. Six of the 10 studies in mature HM reported a positive association between maternal BMI and/or percentage body fat and protein/whey concentration [8,16,28,33,43,49] while three found no association [26,31,47]. Of the two studies in transitional milk, one reported a positive association between these variables [49] while the second found no association [8]. Of the two studies in colostrum, one reported a positive association between HM protein concentration and maternal BMI [8], while the second found no association [31]. Ley 2012 also found no association between maternal BMI and either colostrum or transitional HM [40], and Quinn 2012 also reported no association between maternal BMI or percentage body fat and protein concentrations [44].

### 3.4. Lactose Concentration

**Figure 4:**
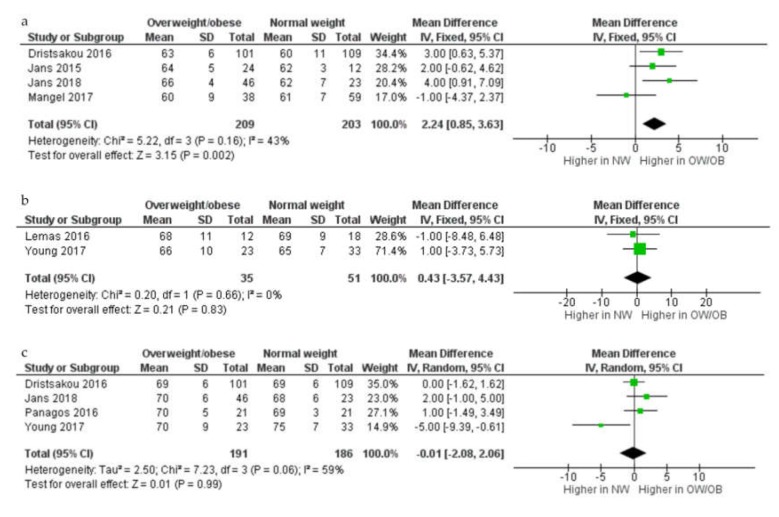
Comparison of lactose concentration (g/L) from normal weight and overweight and obese women according to stage of lactation, colostrum (**a**), transitional (**b**), and mature (**c**). CI, confidence interval; Chi^2^ and I^2^, measures of heterogeneity; IV, inverse variance; NW, normal weight women; OB, obese women; OW, overweight women; SD, standard deviation.

In the meta analyses (*n* = 7; Figure 4), lactose concentration (g/L) in colostrum was higher in overweight and obese compared with normal weight women (MD 2.24 g/L, 95% CI 0.85–3.63, four studies, 209 overweight and obese women and 203 normal weight women). There were, however, no differences seen in lactose concentration in either transitional or mature HM samples between overweight and obese and normal weight women (transitional HM: MD 0.43 g/L, 95% CI –3.57–4.43, two studies, 35 overweight and obese women and 51 normal weight women; mature HM: MD –0.01 g/L, 95% CI –2.08–2.06, random-effects model, *p* = 0.99; I^2^ = 59%; four studies, 191 overweight and obese women and 186 normal weight women).

All four studies not eligible for inclusion in the meta-analysis that compared HM lactose concentration between normal weight and overweight and/or obese women found no difference in lactose concentration between overweight/obese and normal weight women in either transitional (*n* = 1) or mature (*n* = 4) HM (one study reported comparison for more than one HM type) [23,26,27,29], however this included one study in which a BMI >23 kg/m^2^ was classified as overweight an obese. 

Of the nine studies that assessed associations between maternal BMI and/or percentage body fat and lactose concentration in mature HM, two reported a negative association between lactose concentration and maternal BMI [8,49], while six found no association with either maternal BMI or body fat [16,25,26,28,43,47]. Of those studies assessing mature milk, one study reported a negative association between maternal BMI and lactose concentrations in transitional milk [49], while the other found no association for colostrum and transitional milk [8]. In addition, one study that assessed lactose concentrations across lactation (with no distinction between milk type) reported a negative association with maternal BMI [44].

### 3.5. Quality Assessment

The quality assessment is outlined in Table 4 with overall quality of the studies being relatively low. The ability to control for confounders and to state infant’s feeding mode were the categories that scored the lowest across the studies.

Women with pre-existing conditions known to influence HM macronutrient composition, including GDM and diabetes, were excluded in most of the studies (*n* = 18) [23,24,26,28,29,31,34,35,36,39,40,42,43,45,46,47,48,50]. Two studies included women with GDM [30,32], while whether or not women with these conditions were included was unclear [27,49] or not stated [8,9,16,25,33,37,38,41,44] in 11 studies.

## 4. Discussion

This paper describes the first (to our knowledge) systematic synthesis of the current literature investigating the effect of maternal overweight and obesity on HM macronutrient levels. Overall, the meta-analysis indicated that there were differences in the concentrations of fat and lactose in HM of women who were overweight or obese, however the direction of change was dependent on the stage of lactation. While the qualitative analyses broadly supported the findings of the meta-analysis, there was considerable variability, making it difficult to draw robust conclusions.

The higher fat concentration of mature milk in women who were overweight and obese was the most consistent observation across studies. In addition, the majority of studies that examined associations also found a positive correlation between maternal BMI and/or fat mass and HM fat concentration, providing further support of higher levels of fat in HM in women with a higher BMI. The fat concentration of HM is known to be highly dynamic, varying both across the day and even within a feed [51,52], however the role of external factors in regulating fat synthesis in the breast are not as well understood [43]. It is possible that the metabolic dysregulation commonly reported in women with a higher maternal BMI/fat mass, in particular dyslipidemia and higher circulating triglyceride levels, was associated with an increase in the fat concentration of HM [32]. An alternate possibility is that the higher fat concentration was due to higher dietary fat and/or protein intakes in women who were overweight and obese. A Korean study (*n* = 238 women) demonstrated that fat levels in breast milk and maternal diet were highly correlated, and higher fat intakes are commonly reported in association with overweight and obesity [53]. In the DARLING study, a landmark study assessing determinants of macronutrients in HM across the first 12 months, maternal protein intake was also positively related to HM lipid concentration after, but not before, 16 weeks postpartum [43]. Irrespective of the cause of the higher fat concentration, there is the potential for this to impact on infant growth/body composition and long-term obesity risk. A study in the UK involving 614 mother-infant dyads reported that fat percentage in mature HM was inversely correlated with increases in body weight, BMI, and adiposity between 3 and 12 months, and with infant BMI and adiposity at 12 months postpartum [11].

The meta-analysis indicated that the fat concentration in transitional milk was lower in overweight and obese compared to normal weight women. It is important to note, however, that this only included two studies (86 participants) and was not supported by the findings of the studies included in the qualitative analysis. In addition, no impact of maternal overweight and obesity on the fat concentration of colostrum was identified. The fact that maternal overweight and obesity had less impact on HM fat concentration at earlier stages of lactation is consistent with the findings of the DARLING study [43], which indicated that milk composition is more sensitive to maternal factors, including body weight, body composition, and diet later in lactation than during the first few months postpartum. It may be that the rapid mobilization of pregnancy fat stores and dynamic shifts in HM composition that occur in the first few days to weeks postpartum, make it difficult to detect the influence of other external factors on HM composition until later in lactation [43].

The impact of maternal overweight and obesity on HM protein concentration remains inconclusive; while the meta-analysis suggested that there were no differences in HM protein at any lactation stage, some studies from the qualitative synthesis reported a positive association between HM protein and maternal BMI and fat mass across lactation. Protein concentration in HM has previously been shown to decrease progressively across lactation [54,55,56], and studies have also suggested influences of several other factors, including the infant’s gestational age [42], maternal smoking [57], parity [58] and mode of delivery [59], on HM composition. As a result, differences in these factors between studies may have accounted for some of the variability in findings. In previous studies, obesity has been associated with impairments in amino acid metabolism [60], and with elevated amino acid concentrations in both the circulation [61] and HM [62,63]. This raises the possibility that the metabolic changes associated with obesity may affect HM composition, however, there is currently insufficient evidence to draw robust conclusions and further studies are required [64,65,66]. Such studies are of particular relevance given the important role that protein concentrations in HM have in programming of metabolism and growth in early life [67]. Thus, previous studies have reported that HM protein concentrations in mature milk are positively correlated with birth weight [30] and infant BMI at 12 months postpartum [11], while a study in Australia with 20 breastfeeding dyads showed that both HM casein concentration and calculated daily intake of casein in the infant (based on HM concentration and infant milk intake) were positively associated with fat mass and fat mass index, and lower fat-free mass, in the infant at 5, 9, and 12 months postpartum [33].

The concentration of lactose, the major carbohydrate in HM [21], did not appear to be influenced by maternal overweight and obesity in mature or transitional HM. Lactose is known to maintain adequate osmotic pressure in HM [68], and therefore maintaining consistent levels of this component may be important so as not to disrupt this vital function [16]. There was some evidence, albeit limited, that lactose concentrations in colostrum were higher in women who were overweight and obese, however, given the low number of studies and inconsistencies between studies included in the qualitative analysis, it is difficult to draw clear conclusions. Differences is HM lactose concentration in colostrum could potentially be explained by differences in maternal dietary intake, but this seems unlikely given that no previous studies have shown any associations between HM lactose concentrations and maternal diet [43]. 

The major strength of this manuscript is the inclusion of a meta-analysis which enabled us to quantitatively synthesize much of the available evidence. Other strengths of this manuscript are the comprehensive literature search, which included different study designs (both observational and interventional), lactating women from a wide range of countries, both urban and rural areas, a broad range of ethnic and sociodemographic backgrounds, and the ability to evaluate relationships at different lactation stages (colostrum, transitional, and mature HM). In addition, eligibility criteria were applied to ensure that a number of critical elements were reported, i.e., when HM samples were collected (stage of lactation) and when maternal obesity measurements were performed/collected (pre-pregnancy or time of HM collection). Further, the inclusion of other measurements of maternal obesity besides BMI, in particular assessment of body fat by BIA, enabled us to examine relationships between HM macronutrient concentrations and more direct measures of maternal adiposity, although it should be noted that relatively few studies had undertaken such measures. 

Despite these strengths, there were also limitations, the most significant being the small number of studies eligible for both the quantitative and qualitative analyses. Further to this, the quality of the included studies was generally low, and there was a lack of standardization of collection methods and procedures. Physiological and methodological aspects of HM, such as gestational age, method, and time of collection, can significantly influence macronutrient levels [51,52,69] and therefore affect the results of comparisons/associations in individual studies. For instance, fat concentration in HM increases significantly (up to three-fold) from the beginning to the end of a breastfeed or expression [70]. In addition, HM composition varies between preterm and term births [69], and whether the relationship between maternal overweight and/or obesity and HM composition could potentially vary in HM from mothers of term and preterm infants is unknown. In addition, the fact that lactating women from overweight and obese groups may also have other metabolic conditions, such as gestational or pre-gestational diabetes, could influence the results, given emerging evidence of differences in HM composition between women with and without diabetes [71]. Almost all studies utilized BMI to classify women as overweight and obese. While this is a practical measure, it does not always accurately reflect maternal fat mass/body composition, and there is evidence that correlations between some HM components and maternal obesity measures may be stronger for fat mass than for BMI [16]. Further, for the majority of studies reporting associations/correlations between maternal obesity and HM macronutrient concentration, most participants were in the normal BMI range, which may have limited the range of values for assessing correlations, and therefore the results of the correlation analyses. We identified some studies that used a BMI classification not consistent with the WHO guidelines, making it difficult to compare with other studies. The timing of the maternal obesity measurement also varied between studies (i.e., measurements obtained either pre-pregnancy or postpartum), and this may have affected the relationships, since maternal weight (and therefore BMI) may be considerably higher postpartum compared to prior to pregnancy. 

## 5. Conclusions

In summary, our meta-analysis of existing studies has suggested that measures of maternal obesity were associated with changes in fat and lactose concentrations in HM at different lactation stages, whereas there did not appear to be any influence of maternal BMI and/or obesity measures on HM protein concentration. However, the overall quality of the evidence in this area is relatively low and more high-quality studies are needed to better understand the relationships between maternal adiposity and HM composition. This is particularly relevant considering the potential implications of higher HM fat concentration on growth and fat deposition during the first few months of infancy, and long-term BMI and adiposity. The mechanisms through which maternal overweight and obesity can influence HM composition, i.e., as a consequence of maternal metabolic disturbances and/or dietary factors, also remains unclear and further studies are required to investigate this.

## Figures and Tables

**Table 1:** Summary of studies of human milk macronutrient composition included in the systematic review ^1^.

Author, Year	Site	Study Design	Sample Size	Stage of Lactation	Measure Maternal Obesity	Nutrients Assessed		
						Milk Fat or TGs	Milk Protein	Milk Lactose or Carbohydrates
Aleali, 2018 [23]	Iran	Longitudinal	51	1, 2, 3, and 4 wk	BMI	√	√	√
Antonakou, 2013 [24]	Greece	Longitudinal	64	1, 3, and 6 mo	BMI	√		
Aumeistere, 2017 [25]	Latvia	Cross-sectional	28	2–21 mo	BMI			√
Barbosa, 1997 [26]	Mexico	Longitudinal	40	3 and 6 mo	BMI	√	√	√
Brown, 1986 [27]	Bangladesh	Intervention	58	1–9 mo	Arm circumference, skinfold	√	√	√
Bzikowska-Jura, 2018 [28]	Poland	Longitudinal	40	1, 3, and 6 mo	BMI, BIA	√	√	√
Chang, 2015 [8]	Korea	Cross-sectional	2,632	0–8 mo	BMI	√	√	√
DeLuca, 2016 [29]	France	Cross-sectional	100	1 mo	BMI	√	√	√
Dritsakou, 2017 [30]	Greece	Longitudinal	305	3, 7, and 30 day	BMI	√	√	√
Eilers, 2011 [31]	Germany	Longitudinal	77	3 and 28 day	BMI	√	√	
Fujimori, 2015 [32]	Brazil	Cross-sectional	68	2–3 day	BMI	√	√	
Gridneva, 2018 [33]	Australia	Longitudinal	20	2, 5, 9, and 12 mo	BMI, BIS		√	
Jans, 2015 [34]	Belgium	Cross-sectional	48	4 day	BMI	√	√	√
Jans, 2018 [35]	Belgium	Longitudinal	75	3 or 4 day and 1–6 wk	BMI	√	√	√
Kierson, 2006 [36]	USA	Cross-sectional	20	7–21 day	BMI	√		
Kugananthan, 2017 [16]	Australia	Longitudinal	59	2, 5, 9, and 12 mo	BMI, BIS		√	√
Kurniati, 2016 [37]	Indonesia	Cross-sectional	48	1 mo	BMI, BIA	√		
Kwon, 2017 [38]	USA	Cross-sectional	44	2–14 wk	BMI	√	√	
Lemas, 2016 [39]	USA	Cross-sectional	30	2 wk	BMI	√	√	√
Ley, 2012 [40]	Canada	Longitudinal	170	1 or 7 day and 3 mo	BMI	√	√	
Makela, 2013 [41]	Finland	Cross-sectional	163	3 mo	BMI	√		
Mangel, 2017 [42]	Israel	Cross-sectional	109	1–2 day	BMI	√	√	√
Nommsen, 1991 [43]	USA	Longitudinal	92	3, 6, 9, and 12 mo	Skinfold, % IBW	√	√	√
Panagos, 2016 [9]	USA	Cross-sectional	42	4–10 wk	BMI	√	√	√
Quinn, 2012 [44]	Philippines	Cross-sectional	102	0–18 mo	BMI, skinfold	√	√	√
Rudolph, 2017 [45]	USA	Longitudinal	48	2 wk and 4 mo	BMI	√		
Schueler, 2013 [46]	USA	Cross-sectional	13	29–38 day	BMI, waist circumference, DXA	√		
Villalpando, 1992 [47]	Mexico	Cross-sectional	30	4 or 6 mo	BMI, skinfold	√	√	√
Villalpando, 2001 [48]	Mexico	Intervention	10	5–6 mo	BMI, skinfold	√		
Yang, 2014 [49]	China	Cross-sectional	436	5–11, 12–30, 31–60, 61–120 or 121–240 day	BMI	√	√	√
Young, 2017 [50]	USA	Longitudinal	56	2 wk and 4 mo	BMI	√	√	√

^1^ BIA, bioelectrical impedance analysis; BIS, bioelectrical impedance spectroscopy; BMI, body mass index; DXA, dual-energy X-ray absorptiometry; mo, month(s); wk, week(s); TGs, triglycerides; %IBW, pre-pregnancy percent ideal body weight. The checkmark symbol indicates which macronutrient was assessed by each study.

**Table 2:** Effect of maternal obesity on human milk macronutrient composition: key findings ^1,2^.

	Gestational Age	HM Method of Collection	Milk Type	Collection Time	Analytical Method	Outcomes/Effect
**Comparison of Macronutrients between OW/OB and NW Women**						
Aleali, 2018 [23]	Preterm	NS	Transitional and mature	14:00–16:00	MIRIS analyzer, Sweden	No difference in HM fat, protein or lactose between groups
Brown, 1986 [27]	NS	Full expression	Mature	24 h	Gravimetric method, Kjeldahl method and colorimetry	HM fat was higher in women with higher AC and TCSF. No difference in HM protein or lactose between groups
DeLuca, 2016 [29]	Term	Full expression	Mature	9:00–11:00	MIRIS analyzer, Sweden	No difference in HM fat, protein or lactose between groups
Dritsakou, 2017 * [30]	Preterm and term	Full expression	Colostrum, transitional and mature	24 h	MIRIS analyzer, Sweden	HM fat (colostrum, transitional and mature) was higher in OW/OB women. No difference in HM protein and lactose between groups
Fujimori, 2015 [32]	Term	NS	Colostrum	NS	Creamatocrit, Biuret colorimetric	HM fat was higher in OB women. No difference in HM protein between groups
Jans, 2015* [34]	Term	Mid-feed	Colostrum	NS	MIRIS analyzer, Sweden	HM fat was higher in OB women. No difference in HM protein or lactose
Jans, 2018* [35]	Term	Mid-feed	Colostrum, transitional and mature	NS	MIRIS analyzer, Sweden	No difference in HM fat, protein or lactose between groups
Kwon, 2017* [38]	NS	NS	Mature	NS	Lipid extraction, Kjeldahl method	No difference in HM fat or protein between groups
Lemas, 2016* [39]	Term	Mid-feed	Transitional	Morning	Creamatocrit, Bradford protein assay, colorimetric assay	No difference in HM fat, protein or lactose between groups
Makela, 2013* [41]	NS	Pre-feed	Mature	Morning	Lipid extraction	No difference in HM fat content between groups
Mangel, 2017* [42]	Term	Pre-feed	Colostrum	8am–3pm	MIRIS analyzer, Sweden	No difference in HM fat, protein or lactose between groups
Panagos, 2016* [9]	Term	Full expression	Mature	Morning	Julie Z7 Automatic MilkoScope, Germany	No difference in HM fat, protein or lactose between groups
Rudolph, 2017 [45]	Term	Mid-feed	Transitional and mature	Morning	Creamatocrit	HM fat was higher in OW women
Villalpando, 2001 [48]	NS	Full expression	Mature	10:00, 12:00 and 18:00	Gravimetric method	No difference in HM fat between groups
Young, 2017 * [50]	Term	Full expression	Transitional and mature	10:00-13:00	Creamatocrit, Bradford assay, enzymatic method	HM protein (transitional) and lactose (mature) were lower in OW/OB women. No difference in HM fat between groups
**Correlation between maternal measures of maternal obesity and HM macronutrient concentrations**						
Antonakou, 2013 [24]	Term	Pre-feed	Mature	Morning	Creamatocrit	No correlation between HM fat and BMI
Aumeistere, 2017 [25]	NS	NS	Mature	24 h	HPLC	No correlation between HM lactose and BMI
Bzikowska-Jura, 2018 [28]	Term	Pre- and post-feed	Mature	24 h	MIRIS analyzer, Sweden	HM fat (1 and 6 mo) and protein (3 mo) positively correlated with BMI, and HM protein (3 mo) with % body fat. No correlation of HM lactose with either BMI or %body fat
Chang, 2015 [8]	Term	Full expression	Colostrum, transitional and mature	NS	MilkoScan FT2 Foss Analytical, Denmark	HM protein at 0-1 wk, 3–4, and 4–5 mo, and HM fat at 1–2 wk, 2–3, and 7–8 mo positively correlated with BMI. HM lactose at 4–5 and 6–7 mo was negatively correlated with BMI
Eilers, 2011 [31]	Preterm and term	Pre- and post-feed	Colostrum and mature	16:00–20:00	Creamatocrit, BCA protein assay	No correlation between BMI and HM fat or protein
Gridneva, 2018 [33]	Term	Pre- and post-feed	Mature	NS	Bradford protein assay	No correlation between BMI and HM total protein; whey protein was positively correlated to BMI, fat-free mass, fat-free mass index, and fat mass index
Kierson, 2006 [36]	Preterm and term	Full expression	Transitional and mature	NS	Creamatocrit	HM fat was positively correlated to maternal BMI
Kugananthan, 2017 [16]	Term	Pre- and post-feed	Mature	9:30–11:30	Bradford assay, Enzymatic spectrophotometric method	HM protein positively correlated with %body fat, but not BMI. No correlation of either %body fat or BMI with HM lactose.
Kurniati, 2016 [37]	Term	Mid-feed	Mature	6:00–8:00	Creamatocrit	No correlation between %body fat and HM fat
Ley, 2012 [40]	Term	early milk Full expression	Colostrum/Transitional and mature	NS	Creamatocrit, BCA protein assay	No correlation between BMI and HM fat or protein
Nommsen, 1991 [43]	NS	Full expression	Mature	24 h	Folch extraction, Lowry assay, colorimetric assay	HM fat (6, 9, and 12 mo) and protein (9 mo) positively correlated with %IBM. No correlations with HM lactose
Quinn, 2012 [44]	NS	Mid-feed	Colostrum, transitional and mature	6:00–10:30	Rose-Gottlieb extraction, automated analyzer, phenol- sulfuric acid method	HM lactose was inversely correlated with BMI. No relationship between %body fat and HM fat, protein, or lactose
Schueler, 2013 [46]	NS	Pre- and post-feed	Mature	7:00-10:00	Creamatocrit	HM fat positively correlated with total fat mass, BMI, body weight, and %body fat
Villalpando, 1992 [47]	Term	Full expression	Mature	10:00, 12:00, and 18:00	Jeejeebhoy method, Kjeldahl method, automated enzymatic method	HM fat positively correlated with body weight, BMI, and %body fat. No associations between these measures and HM protein or lactose
Yang, 2014 [49]	Term	Full expression	Transitional and mature	9:00–11:00	MIRIS analyzer, Sweden	HM protein and fat were positively correlated, and HM lactose negatively correlated, with BMI
**Both comparison and correlation**						
Barbosa, 1997 [26]	Term	Full expression	Mature	10:00, 14:00, and 18:00	Gravimetric method, Kjeldahl method, automatic enzyme method	HM fat was positively correlated with BMI and %body fat, and was lower in the lower BMI group (<23 kg/m^2^) compared to the higher (≥23 kg/m^2^). HM protein and lactose were not correlated with BMI or %body fat and no different between these BMI groups

^1^ Analytical methods are reported based on the sequence noted on outcomes, otherwise reporting follows the “fat, protein and lactose” sequence. Maternal obesity values are reported as mean ± SD or mean (range) when SD is not available. Studies included in the meta-analysis are indicated with *. ^2^ Symbol—was used to represent information not stated or unclear. AC, Arm circumference; BCA, Bicinchoninic acid method; BMI, body mass index; HM, human milk; HPLC, high-performance liquid chromatography; % IBM, pre-pregnancy percent ideal body weight; h, hour; MIRIS, Mid-infrared milk analyzer; mo, month (s); NS, not stated; NW, normal weight; OB, obese; OW, overweight; PT, preterm; T, term; TCSF, triceps skinfold thickness; wk, week (s).

**Table 3:** Summary results of qualitative synthesis across all stages of lactation for studies reporting both comparison and correlation analysis ^1^.

Milk Macronutrient	Effect									
	Studies, *n*	Positive association with Maternal BMI/Adiposity			No Association with Maternal BMI/Adiposity			Negative Association with Maternal BMI/Adiposity		
		All	Comparison	Correlation	All	Comparison	Correlation	All	Comparison	Correlation
Fat	19	11	4	8	8	3	5	0	0	0
Protein	15	6	0	6	9	5	5	0	0	0
Lactose	12	0	0	0	9	4	6	3	0	3

^1^ BMI, body mass index; n, total number of included studies. “All” combines the number of studies from both comparison and correlation analysis. One study has reported both comparison and correlation analysis [26] which influenced on the number of studies underlined.

**Table 4:** Quality and reporting assessment for studies measuring macronutrient concentrations in human milk ^1^.

Reference	Representativeness Cohort	Measure of Maternal Obesity	Controls for Confounders	Sample Size (Small, Medium or Large Study)	State Gestational Age	State Feeding Mode	Standard Time of HM Collection	State HM Collection Method
Aleali, 2018 [23]	X	√	x	Medium	√	x	√	x
Antonakou, 2013 [24]	√	√	√	Medium	√	√	√	√
Aumeistere, 2017 [25]	X	x	x	Small	x	√	√	x
Barbosa, 1997 [26]	X	√	x	Small	√	x	√	√
Brown, 1986 [27]	√	√	√	Medium	x	x	√	√
Bzikowska-Jura, 2018 [28]	√	√	x	Small	√	√	√	√
Chang, 2015 [8]	√	√	√	Large	√	x	x	√
DeLuca, 2016 [29]	√	√	√	Medium	√	√	√	√
Dritsakou, 2017 [30]	X	x	x	Large	√	√	√	√
Eilers, 2011 [31]	√	x	x	Medium	√	x	√	√
Fujimori, 2015 [32]	√	x	x	Medium	√	√	x	x
Gridneva, 2018 [33]	√	√	√	Small	√	√	x	√
Jans, 2015 [34]	√	√	x	Small	√	x	x	√
Jans, 2018 [35]	√	√	x	Medium	√	x	x	√
Kierson, 2006 [36]	X	x	x	Small	√	x	x	√
Kugananthan, 2017 [16]	√	√	x	Medium	√	√	√	√
Kurniati, 2016 [37]	√	√	x	Small	√	√	√	√
Kwon, 2017 [38]	√	x	x	Small	x	x	x	x
Lemas, 2016 [39]	√	√	√	Small	√	√	√	√
Ley, 2012 [40]	√	x	√	Large	√	x	x	x
Makela, 2013 [41]	√	√	x	Large	x	x	√	√
Mangel, 2017 [42]	√	x	x	Large	√	x	√	√
Nommsen, 1991 [43]	√	√	√	Medium	x	√	√	√
Panagos, 2016 [9]	√	√	√	Small	√	x	√	√
Quinn, 2012 [44]	√	√	√	Large	x	√	√	√
Rudolph, 2017 [45]	√	x	x	Small	√	√	√	√
Schueler, 2013 [46]	√	√	√	Small	x	√	√	√
Villalpando, 1992 [47]	X	√	x	Small	√	√	√	√
Villalpando, 2001 [48]	X	√	x	Small	x	√	√	√
Yang, 2014 [49]	√	√	√	Large	√	√	√	√
Young, 2017 [50]	√	√	√	Medium	√	√	√	√

^1^ Sample size is defined as small = studies with <50 participants; medium = studies with between 50–100 participants; and large = studies with >100 participants. The checkmark symbol indicates yes and cross symbol indicates no.
